# Report of a recurrent cerebral venous thrombosis in a young athlete

**DOI:** 10.1186/s12883-014-0182-3

**Published:** 2014-09-22

**Authors:** Sébastien Richard, Jean-Christophe Lacour, Birgit Frotscher, Ana Enea, Gioia Mione, Xavier Ducrocq

**Affiliations:** Department of Neurology, University Hospital of Nancy, Hopital Central, 29 avenue du Marechal de Lattre de Tassigny-CO n, 34 54035 Nancy Cedex, France; Laboratory of Haematology, University Hospital of Nancy, Hôpitaux de Brabois, rue du Morvan, 54511 Vandoeuvre-lès-Nancy Cedex, France

**Keywords:** Cerebral venous thrombosis, Exercise training, Athlete, Sport

## Abstract

**Background:**

Reports of occurrence of deep vein thrombosis during intensive sport are scarce. While a few cases have been described in the cerebral territory, these are only in the context of traumatism or anabolic agent consumption. Thus, causality with exercise remains uncertain and the mechanisms hypothetic. We present the case of a young athlete who experienced two episodes of severe cerebral venous thromboses (CVT), both during intensive training, in the absence of any other known thrombogenic factor.

**Case presentation:**

A healthy 26-year-old man presented a thrombosis of the superior sagittal sinus during recent intensive training for a triathlon. Investigation at the time found no drug or anabolic steroid consumption, or any hematologic or coagulation disturbance. Anticoagulation therapy was initiated for 10 months with good outcome. One year later, soon after returning to intensive exercise, mainly running, the patient presented a thrombosis of the straight sinus complicated by bithalamic hyperintensities observed on T2 magnetic resonance imaging sequences. Anticoagulation treatment was reinitiated and led to repermeabilization of the cerebral vein and reversibility of thalamic abnormalities. Four months later, the patient was free of headache and had no cognitive impairment. He continues to practice intensive sport with vitamin K antagonist as preventive treatment.

**Conclusion:**

This is the first case report of recurrent CVT in a context of intensive sport, without any other thrombogenic features, suggesting a causal link. Intensive exercise should be considered as a potential promoting factor of CVT and investigated during routine examination.

## Background

Cerebral venous thrombosis (CVT) is an uncommon condition that can be caused by a variety of factors including thrombophilia, pregnancy, puerperium, infection and malignancy. It is important to establish the cause of CVT to determine optimal long term preventive treatment and help the patient avoid future thrombogenic conditions. However, in around 12% of cases [1] the cause remains unknown. Reports of venous thromboembolism occurring in athletes, especially with an intracranial location, are few and far between [2]. Furthermore, previous reports have been associated with thrombogenic factors like traumatism [3] or anabolic agent consumption [4]. We present the case of a young athlete who experienced two separate episodes of CVT, each occurring during intensive training for a triathlon.

## Case presentation

**Figure 1:**
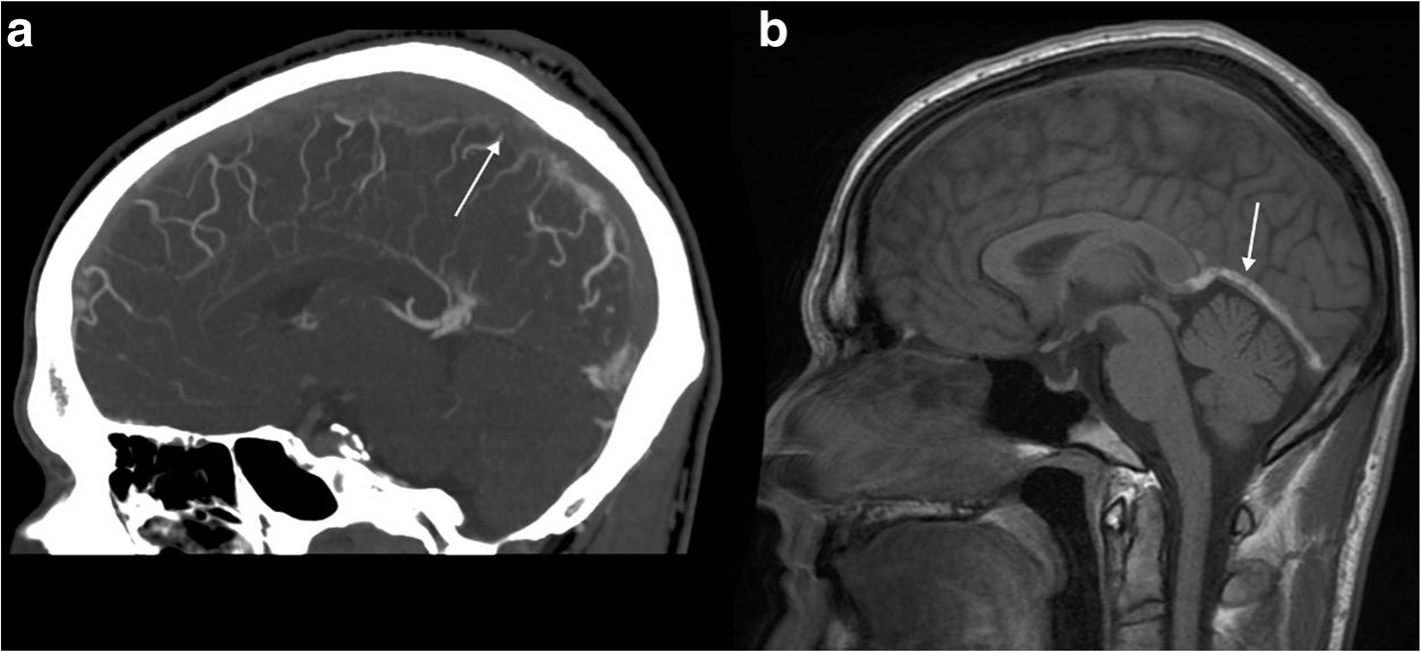
**Computed tomography angiography (a) showing an extended superior sagittal sinus thrombosis (arrow) and T1 weighted sagittal magnetic resonance imaging (b) showing thrombosis of the straight sinus (arrow).**

**Figure 2:**
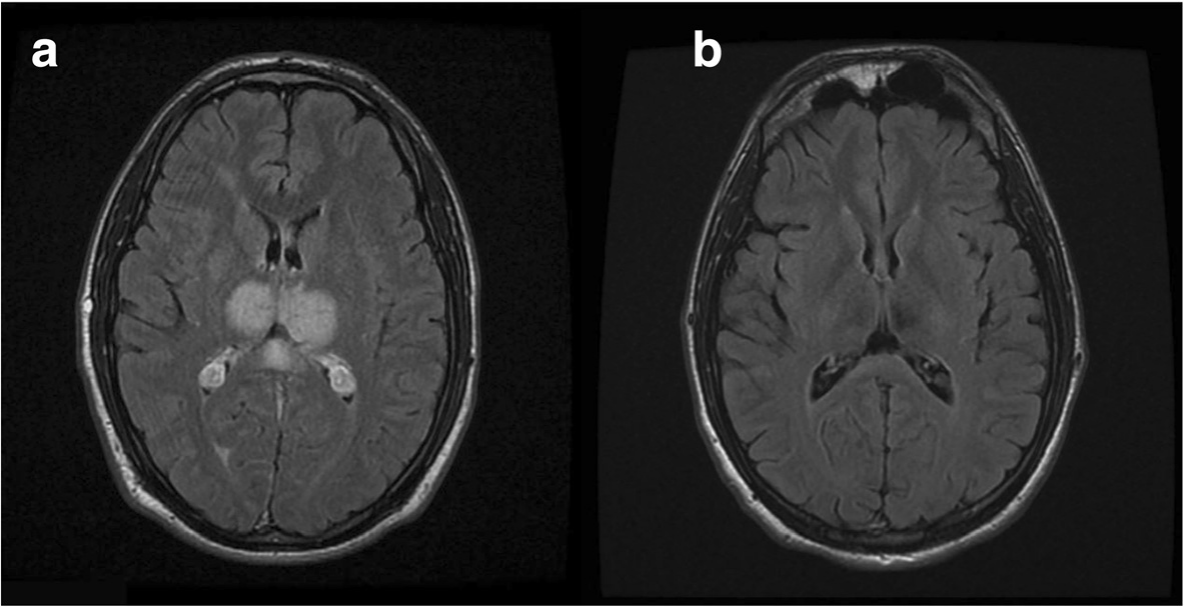
**Fluid attenuated inversion recovery axial magnetic resonance imaging showing bithalamic hyperintensities (a), reversible with anticoagulation therapy (b).**

A healthy and athletic 26-year-old man was hospitalized in January 2012 because of progressive headache with vomiting and diplopia. Ophthalmic examination showed a bilateral sixth nerve palsy and papilledema. Computed tomography (CT) angiography revealed an extended superior sagittal sinus thrombosis (Figure 1a). A detailed interview did not find any personal or family medical history of vascular disease, or any drug consumption or androgenic anabolic therapy. However, it emerged that the patient had been training intensively for about 15 hours a week for 6 months – mainly running but also cycling and swimming – to prepare for his first triathlon and several running races. Before this period of intensive training, he used to run for no more than 2 hours a week. Blood tests showed a hemoglobin level of 15 g/dL, platelet count at 260 G/L without JAK2 V617F mutation, C-reactive protein level under 5 mg/L, homocystein level of 9 μmol/L. All the coagulation tests – prothrombin time, activated partial thromboplastin time, antithrombin, protein C, protein S, activated protein C resistance, mutation of prothrombin gene, lupus anticoagulant and anticardiolipin antibody – were normal. An intravenous treatment of unfractionated heparin was initiated with an early overlap to vitamin K antagonists (VKA). Ten months later, in October 2012, clinical examination was normal and the patient was headache free. A control magnetic resonance imaging (MRI) showed a near complete resolution and anticoagulation therapy was discontinued. The patient began intensive training again consisting of about 10 hours of running a week. However, several weeks later he complained of progressive headache and cognitive impairment leading to another hospitalization in December 2012. Brain MRI revealed thrombosis of the straight sinus extended to the right lateral sinus (Figure 1b) with bithalamic hyperintensities observed in T2 and fluid attenuated inversion recovery sequences (Figure 2a), and no modification of the aspect of the superior sagittal sinus. There was no change in the hemoglobin level or platelet count. We ran the same coagulation tests as for the first episode completed by a test for anti-β2-glycoprotein 1 antibodies for antiphospholipid syndrome. All tests were negative once again. The absence of intravascular hemolysis signs and a normal flow cytometry ruled out paroxysmal noctural hemoglobinuria. Time-resolved imaging of contrast kinetics MR angiography did not reveal any cerebral arteriovenous malformation. A thoraco-abdomino-pelvic CT scan was performed to investigate for cancer and also turned out to be normal. Anticoagulant therapy, with unfractionated heparin and then VKA, was resumed. In May 2013, the patient no longer suffered from headaches or cognitive impairment. Brain MRI showed a complete repermeabilization of the straight and right lateral sinus, and reversion of thalamic abnormalities (Figure 2b). To date, the patient is still on VKA and has resumed intensive training. He has also participated in another triathlon without any complications despite a bicycle fall.

## Discussion

CVT following long endurance exercise without traumatism or consumption of anabolic substances has never been reported before. The few reports of venous thromboembolism occurring during a sports activity nearly always involve long strenuous running as in a marathon. Most cases report deep vein thrombosis of the lower limbs and pulmonary embolism [2], and the patients present other risk factors for thrombosis such as oral oestroprogestative contraception or a defect of the anticoagulant system rendering causality with the practice of sport uncertain [5]. Cases in the cerebral territory have been described in a context of traumatism [3] or anabolic agent consumption [4]. In addition, many athletes travel before exercise which increases the risk of venous thrombosis. The underlying mechanism by which sport could cause venous thromboembolism is difficult to establish even though elevated venous thrombotic risk markers like D-dimer, microparticles and p-selectin have been observed in athletes after a marathon [6]. However, it is thought that components of Virchow’s triad could be involved. Dehydration appears to be the most likely cause leading to hemorheological disturbances due to increased blood viscosity, high hematocrit and red cell aggregation [7,8]. High pressure in the venous territory may cause turbulence and reduced blood flow [2]. A defective fibrinolytic system, mainly occurring during recovery, along with increased thrombin and fibrin synthesis and a high platelet count and activity have also been suggested to play a role [9,10]. Finally, traumatism of the vessel wall may be responsible for activation of factor X which could initiate thrombosis [2].

Several factors support the hypothesis of causality in the case we describe here. The patient presented two CVTs in two different locations. Symptoms of both CVTs occbucurred during several weeks of intensive training, mainly running. Repeated thorough examination for thrombogenic risk factors did not reveal any platelet or coagulation disorders. An important point to note in this case is that both CVT episodes occurred in the intracranial territory. Intracranial sinuses do not have valves. This allows the blood to circulate in both directions but may fail to ensure blood flow velocity in some cases and favour thrombosis. An increase in pressure in the venous system coupled with dehydration caused by sustained effort could reduce intracranial venous return, inducing a decrease in blood flow in the intracranial vein and triggering thrombosis. In CVT, the development of the thrombus may be subacute and symptoms delayed. As we saw the patient several days after he had competed, our initial assessment cannot rule out severe dehydration following exercise as being the cause of CVT. However, this explanation alone is not satisfactory and the nature of the case suggests a possible combination of features including an unidentified congenital or acquired thrombogenic factor. Another important question this case raises is the long term anticoagulant treatment for a patient who intends to continue practicing intensive sport. While VKA treatment is justified by the two severe CVTs, there is a high risk of hemorrhagic complications as athletes are exposed to accidents and traumatism.

## Conclusion

This is the first report of recurrent CVT in an athlete without other known thrombogenic features suggesting that extreme exercise could trigger CVT in predisposed patients. Even if the mechanisms are not entirely understood, routine investigation to determine the cause of a CVT should include asking the patient about any long strenuous sport exercise preceding the occurrence of the first symptoms.

## Consent

Written informed consent was obtained from the patient for publication of this Case report and any accompanying images. A copy of the written consent is available for review by the series editor of this journal.

## Abbreviations

CVT: Cerebral venous thrombosis
CT: Computed tomography
VKA: Vitamin K antagonists
MRI: Magnetic resonance imaging
